# Using a modified nominal group technique to develop general practice

**DOI:** 10.1186/s12875-018-0811-9

**Published:** 2018-07-18

**Authors:** Elisabeth Søndergaard, Ruth K. Ertmann, Susanne Reventlow, Kirsten Lykke

**Affiliations:** The Research Unit for General Practice and Section of General Practice, Department of Public Health, University of Copenhagen, Copenhagen, Denmark

**Keywords:** Consensus methods, Nominal group technique, Organisational development, General practice, Primary health care, Qualitative research, Denmark, Electronic health records

## Abstract

**Background:**

There are few areas of health care where sufficient research-based evidence exists and primary health care is no exception. In the absence of such evidence, the development of assisted support must be based on the opinions and experience of professionals with knowledge of the relevant field. The purpose of this research project is to explore how the nominal group technique can be used to establish consensus by analysing how it supported the development of structured, knowledge-based, electronic health records for preventive child health examinations in Danish general practice.

**Methods:**

We convened an expert panel of five general practitioners with a special interest in the preventive child health examinations. We introduced the panel to the nominal group technique, a well-established, structured, multistep, facilitated, group meeting technique used to generate consensus. The panel used the technique to agree on the key clinical and socioeconomic themes to include in new electronic records for the seven preventive child health examinations in Denmark. The panel met three times over a four-month period between 2013 and 2014 and their meetings lasted between two-and-a-half and five hours.

**Results:**

1) The structured and stepwise process of the nominal group technique supported our expert panel’s focus as well as their equal opportunities to speak. 2) The method’s flexibility enabled participants to work as a group and in pairs to discuss and refine thematic classifications. 3) Serial meetings supported continual evaluation, critical reflection, and knowledge searches, enabling our panel to produce a template that could be adapted for all seven preventive child health examinations.

**Conclusion:**

The nominal group technique proved to be a useful method for reaching consensus by identifying key quality markers for use in daily clinical practice. Our study focused on the development of content and a layout for systematic, knowledge-based, electronic health records. We recommend the method as a suitable working tool for dealing with complex questions in general practice or similar settings, and we present and discuss modifications to the original model.

## Background

General practitioners (GPs) regularly make difficult choices about treatment options. Guidelines are one way of assisting GPs in decision-making and, in an ideal world, guidelines would be based on evidence derived from rigorously conducted empirical studies. In practice, there are few areas of health care where sufficient research-based evidence exists or can even be produced [1], and this is especially so within primary healthcare [2]. In such situations, the development of assisted support will inevitably be based, largely or in part, on the opinions and experience of clinicians and others with knowledge of the relevant field [3].

Since 2013, a group of Danish GPs has worked on producing systematic and knowledge-based electronic health records for the seven preventive child health examinations (PCHEs) held in general practice.

The Danish National Board of Health provides guidelines for PCHEs, but the recommendations are extensive and cover all aspects of a child’s health [4]. There is no structured and systematic process in place to determine which of the comprehensive recommendations is the most important to focus on during the limited time available to carry out the PCHEs, nor is there a nationally aligned process for keeping journal notes. The vision is to make new electronic journal records available to all Danish GPs and the idea is that GPs’ use of an electronic health record, with its potential for decision support and easy access to previous findings, will support their work and make it easier to keep an overview of the patient’s case history.^1^ The development of electronic records therefore holds the potential for a quality development in child healthcare in Denmark.

Given the likely diversity of opinion that any group of people may display when considering a topic, formalised methods, such as consensus techniques, are essential for organising subjective judgments in group work. Consensus techniques have been successfully used by several research groups in their work to develop quality markers in complex clinical areas, such as angina [5], emergency care [6], cancer [7], and also within the field of child healthcare [8–10].

The three most common consensus methods used for medical and health services research are the Delphi method, the consensus development conference, and the nominal group technique (NGT) [11]. The Delphi method is a forecasting method based on several rounds of questionnaires sent to a panel of experts. The anonymous, written responses are aggregated and shared with the group after each round [12]. The consensus development conference brings together practitioners, researchers, and consumers over a period of several days to seek general agreement, or consensus, on the efficacy, safety, and appropriate conditions for the use of various medical and surgical procedures, drugs, and devices [13]. The third method, and the one we selected in the present study, is the NGT.

The NGT is a structured, well-established, multistep, facilitated, group meeting technique used to generate and prioritise responses to a specific question by a group of people who have expert insight into a particular area of interest [2, 11, 14, 15]. It is an organised process that gives participants an equal opportunity to contribute their personal views before inviting them to build on the reflections of others to develop their own thoughts, and finally to reach consensus about the issues raised in the original question [8]. The NGT has been applied on several occasions for projects in general practice [16–19]. It has an advantage in that its format resembles the way Danish GPs are accustomed to collaborating in network groups, where experiences and challenges from everyday working life in practice are shared and discussed [20].

In this study the NGT was used with a twofold purpose. First, it was a way to systematise and develop the content of PCHEs; and second, it was a method to develop the format of electronic health records to be used in PCHEs. These two parallel purposes were strongly interlinked.

To bridge the gap between research and practice, evidence as well as its applicability should be considered when formulating recommendations. It is important that recommendations are compatible with existing norms and values and it is therefore essential that practitioners, in this case the future users of electronic health records, participate in the development of practice [21].

In this paper we explore how the NGT can be used to establish consensus in a complex clinical field by analysing how it supported the development of structured electronic health records for PCHEs in Danish general practice.

## Methods

This study applied group discussions based on the NGT method in an adapted serial meeting design. The adapted design complies with the checklist created by Humphrey-Murto et al. to ensure methodological rigour when using consensus group methods, with only one deviation concerning anonymous re-ranking of feedback [22]. In-depth descriptions of the original steps in the NGT method have been reported extensively elsewhere [8, 23].

The project was conceived and designed by KL and RE, who are both experienced GPs specialising in research on children’s health. During the meetings, KL was in the facilitator’s role and RE participated as a member of the NGT panel. RE participated on the same terms as the other panel members, meaning that e.g. she waited for her turn to speak in the rounds, and her opinion carried no more weight than any of the other participants. More importantly, RE was aware of her double role in the project and its potential downfalls. This demanded a continuous reflection on her position, which we shall return to in the discussion section.

As well as RE, we purposively identified four GPs known for their broad knowledge and expertise in general practice and their specific interest in the PCHEs. We invited them to constitute the expert panel. Verbal informed consent was given before the four recruited GPs freely and informed chose to participate in the project. All five participating GPs worked either in Region Zealand or in the capital area of Copenhagen in Denmark. Prior to the first meeting the GPs were asked to read the report: *Evaluation of the Preventive Child Consultations in General Practice* [4] and before each meeting they were also asked to read the chapters in the Danish National Board of Health’s guidelines on PCHEs [24] relevant for that particular meeting’s focus. In this way we pursued a systematic method combining evidence and expert opinion.

In addition to background reading, we asked the participating GPs to be extra observant when carrying out PCHEs in the period leading up to the first meeting. We encouraged them to ask parents about their needs and expectations during the PCHEs. Throughout the working period of four months, the expert panel met three times. The meetings lasted five hours, five hours, and two-and-a-half hours respectively, and the four invited participants were offered compensation for their time. All meetings took place at facilities convenient to the practice of two of the participants.

The main aim of NGT is to generate themes and issues, which are discussed and ranked by the group. At the first meeting, KL described the NGT as a method to the panel members who had the opportunity to ask questions. This introduction was a factual description of the method’s different steps and did not have any content or comment that would influence participants and the task in hand. After the introduction, KL asked the panel the nominal question: *What do you consider important to prioritise in the preventive child health examination at five weeks, and what do parents think is important, according to your experience and knowledge*? The question was developed based on KL’s extensive work with the PCHEs [25–27] and RE’s previous experience with developing electronic health records for antenatal care visits in general practice in Denmark. At this stage, the panel was given no guidance on how broad or narrow their focus should be.

The original plan was to work on the first three PCHEs, which take place when a baby is five weeks, five months, and twelve months old; one PCHE per scheduled meeting. However, while working through the steps of the NGT during the first meeting, the group found it necessary to make adjustments and deviations to the original model, outlined by Gallagher et al. [23].

**Fig. 1:**
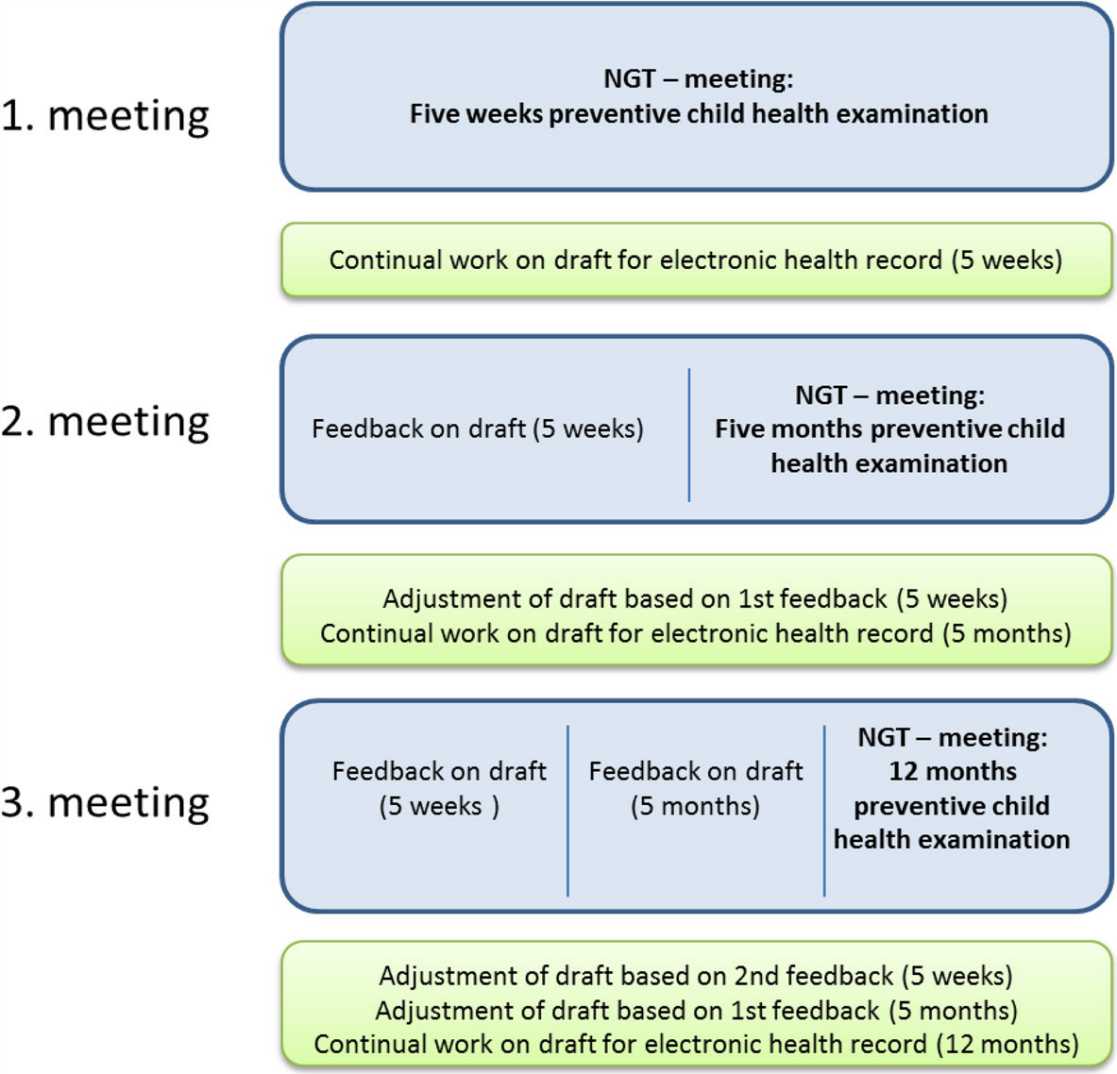
Three NGT meetings were planned from the beginning; one for each of the first three preventive child health examinations in general practice. Between meetings RE and KL continually worked with the document from the previous meeting, which was then discussed, adjusted and approved at the following meeting

The five hours allocated to develop a health record for the first PCHE were not enough to meet the project’s combined objective: exploratory research involving a qualitative understanding of the priorities; and the development of a concrete product in the form of a systematic electronic health record. As a result, it was agreed in plenum that KL and RE should work with the draft produced by the panel between the first and the second meeting. This work solely concerned linguistic and structural aspects and a conscious effort was made to keep the content unchanged. The re-edited draft was then presented to participants at the second meeting, where it was critically evaluated, adjusted, and approved in plenum. In this way, a mutual understanding was secured in a forum in which the participants were both informants and collaborators. This pattern was repeated between the second and third meetings and became the model for working with drafts of the succeeding electronic health records (Fig. 1). Consensus was defined as having been achieved when there were no further comments or suggestions for corrections from any of the participants. Achieved consensus determined the process. The continual and circular re-evaluation of the drafts enhanced the process and secured communicative validity [28].

The group’s experience from working with the content and structure of the first electronic health record was used strategically at the next meeting when the focus shifted to the succeeding PCHE. During the meetings, the atmosphere was jovial and enthusiastic. The participation of KL and RE as the project’s initiators did not seem to affect or influence the four invited GPs. All participants took part in the discussions equally and appeared confident in their roles as well as eager to contribute with their individual perspectives on the work.

In addition to working papers from the meeting rounds and the different draft versions of the electronic health records for the first three PCHEs, material for the present article also consisted of field notes produced during one of the meetings by ES, who participated as an observer. Having an observer in the research project enhanced opportunities for noticing aspects of interpersonal communication and group dynamic that are taken for granted or missed by participants due to their immediate obviousness [29]. The observations and field notes permitted an extra level of abstraction in the discussion of the group’s use of the NGT, particularly with regard to the steps of the model where the group deviated from the original structure.

Based on the output of the meetings, including descriptive field notes, we conducted a thematic text analysis to identify important areas of new knowledge and to better understand what the modified version of the NGT meant for the validity of the method.

## Results

The use of the NGT made it possible to combine idea generation and problem solving as two complementary parts of the same process. This makes the method well suited for development work in general practice with its complex characteristics and demands for applicability. Three main categories of experience were identified and these are described and appraised below. For clarity, the third category is divided into four sub-categories.

### Keeping focus and supporting equal opportunities to speak

The structured and stepwise process of the NGT ensured that the energetic expert panel kept focus on the defined purpose, while the repeated table rounds supported opportunities for participants to be equally heard. The method’s face-to-face approach integrated non-verbal communication, such as laughter; while the structured design minimised potential power structures that can appear when participants already know each other, or when one of the panelists is also the initiator of the project, which was our experience on this project.

### Generating new perspectives on clinical practice

During steps 4 and 5 (Table 1) the participants became aware of the potential to re-use knowledge previously obtained about the patient (Table 2, column 3). Prior to the seven PCHEs, the Danish preventive healthcare programme has three antenatal care visits and, in principle, all ten visits are conducted by the same GP. Data from antenatal care visits are recorded in the mother’s journal, which is not automatically consulted in the PCHEs that follow. The process of the group discussions generated an awareness of the prospective re-use of knowledge gathered during the antenatal care visits, such as the pregnancy’s development or the family’s socioeconomic situation.

**Table 1:** An outline of the steps in the original NGT model and the deviations and attributions made to the structure during the working process with the development of an electronic health record for the examination of babies at 5 weeks

A case study – working with the preventive child health examination at five weeks		
	The original model	Attributions and deviations
Step 1	Introduction	
Step 2	Each individual answers the overall question. Silent generation of ideas in writing.	
Step 3	Table rounds where each participant in turn presents a theme from his/her list. During the presentations, new ideas are generated and rounds continue until all items are listed.	
Step 4	The different themes are discussed and classified. Listing of ideas on flip chart.	The different themes were discussed and organised in categories.
Step 5	Each participant selects 10 of the listed themes in silence. All themes are ranked and given points from 1 to 10. The most important theme receives 10 points.	Working together in pairs the categories were ranged according to the structure of the consultation.
Step 6	Pause, while a prioritised consensus list is produced.	30 min break. No prioritised list produced.
Step 7	The prioritised list is discussed	The thematic categories were presented in plenum and discussed. It was agreed that KL and RE should continue working with the format of the electronic health record in the time until the next meeting.
Step 8	All participants re-evaluate the list. First individually, thereafter in plenum.	Two months’ intermission where KL and RE continually worked on a revised version. The newest version of the electronic health record was discussed and adjusted in plenum at the following meeting. 1-month intermission where the electronic health record was further revised. Final discussions in the group during the next meeting.

**Table 2:** In the first column, all the participants’ ideas are shown in the order they appeared during the table rounds and in the short formulations the proposers found adequate

First rounds of the nominal group process	Clarification and categorisation	After linguistic and structural editing by KL and RE
1. Setting the scene: what, why, how – long process. 2. The parental experience of pregnancy and birth 3. Is there something in particular you would like to discuss? 4. Has the development from birth until now been satisfying? 5. How are things coming along? 6. Did anything happen during birth that you experienced as a threat? 7. How is the family handling the new family member – network? 8. Follow-up from visiting nurse/ place of birth 9. The contact between the GP/mother/child/father 10. Interaction/Attachment 11. The parents’ experience of the parenting role 12. Recognise – Issues with the parents that make me wonder whether this is a vulnerable family 13. Support the parents’ belief in their own capabilities 14. The objective examination 15. The child’s rhythm – sleep patterns, food, crying, bowel function 16. The parents own childhood 17. Preventions themes – smoking, falls, and sleeping positions. 18. Transparency in the examination 19. Conclusion, transparency and follow-up 20. Advice and guidelines concerning a sick child 21. Information for parents prior to the consultation 22. Parental leave – is co-parent at home? childcare, economy 23. To create a feeling of security - future cooperation 24. Family structure 25. Support the parents in their care for their child.	1. Setting the scene At the beginning and at the end of the examination: what, why, how – long process. Vaccinations. What is a preventive child health examination? The goal? Adjust expectations. Transparency in the examinations. Information for parents prior to the consultation. To create a feeling of security - future cooperation 2. The parents’ experience of pregnancy, birth, and the first weeks with the baby Did anything happen during birth that you experienced as a threat? Has the development from birth until this point been satisfying? How are things coming along? Baby blues? (Mother, child, father, the family). 3. Is there something in particular you would like to discuss? The parents’ needs. 4. How is the family handling the new family member? Hard work – siblings – feeling tired – network. The parents’ experience of the parenting role, making ends meet, frustration. Attachment. Parental leave – is co-parent at home – childcare 5. Follow-up from visiting nurse/ place of birth Have they attended the relevant examinations? Have they received answers from tests? 6. Recognise – Issues with the parents that make me wonder whether this is a vulnerable family Economy, resources, education, work. The parents’ experiences. Family structure: single, half-half, donor, adopted, etc. 7. Objective examination. Contact between GP/ mother/child/father interaction. 8. The child’s rhythm Sleep patterns, food, crying, bowel function, well-being. The GP’s evaluation and communication of what is considered as normal. 9. The parents’ own childhood Previous/ongoing family trauma. Preconditions for attachments. 10. Prevention themes Smoking, falls, sleeping positions. Vaccinations. Advice and guidelines concerning a sick child. 11. Conclusion Reciprocity. Support the parents’ belief in their own capabilities Follow-up, appointments. Transparency	Information folder (Could we ask the parents a few questions at the same time?) Knowledge previously collected from the antenatal care visits: The name of the child’s father and CPR number. The siblings’ names and CPRs. Relationship status. Chronic diseases among mother or father. Mother’s/father’s work title, place of birth. Mother’s/father’s potential threatening social or emotional condition. 1. Did the parents have anything particular they wanted to talk with the GP about? Yes _____ No ________ 2. Follow-up on information transferred from the antenatal care visits, has anything changed? Yes ____ No ________ 3. The parents’ experience of pregnancy and birth 3.1 Follow-up on pregnancy. Certain experiences/worries? Yes ______ No ______ 3.2 Follow-up on birth Certain experiences/worries? Yes ______ No ______ 3.3 Is contact with a visiting nurse established? Does the visiting nurse have any wishes for themes that should be discussed at the PCHE? Yes ______ No_______ 3.4 Has the child had a PKU-test? Yes _____ No ________ Hearing screening test: Yes ______ No _______ Remarks: ______________ 4. Parents’ experience of the first weeks 4.1 How are things coming along? (Mother, father, sibling, family, handling new tasks). 4.2 Are mother and child gaining a common rhythm? Yes ______ No ________ (Sleep, meals, bowel functions, can the child be comforted when crying? Do mother and father feel they can cope?). 4.3 Breast-feeding Yes _____ No ______ Partly_______ 5. Objective examination (Specified in 16 items/points following the guidelines from The National Board of Health). 6. Discussed birth control Yes, themes:___ No ____ 7. Conclusion An overall estimation of the child’s wellbeing and the family’s resources and risks. 8. Follow-up Yes ______ No ______ E.g. an extra consultation at the GP or a reference to a specialist/or the social system.

In the second column, all the ideas have been elaborated by the original proposer and the group has, both jointly and in pairs, organised the many ideas into categories. The third column shows the final version of both content and structure of the systematic health record. This was completed after discussion and editing at the third and last meeting. This proposal has subsequently been edited into an electronic format which is not shown in this article

### Flexibility of the NGT model

The NGT proved to be a highly flexible model well suited to the complex research question we asked, and conducive to detailed discussion and elucidation of themes and issues.

#### Discussions and thematic classification in pairs

The panel recognised early in the working process that it would not be favourable to strictly follow the model’s original outline. For example, the discussions and clarifications carried out in step 4 (Table 1) revealed that it did not make sense to produce a prioritised list, as the model prescribes. Since all the suggested themes were important, the expert panel found it more relevant to organise them into broader thematic categories and line them up in that way. The work with these categories was carried out first as a group and then the panel divided into pairs to further discuss the categories. This resulted in an outline of the first draft of the electronic health record (Table 2, column 2).

#### Serial meetings

Serial meetings provided time for continual evaluation and the search for more information.

The original time allocated to work with electronic health records turned out to be too short to produce adequate content and a format for each record. As a consequence, drafts were linguistically and structurally reorganised by KL and RE between meetings (Fig. 1). At the same time, participants had opportunities to test in their practices aspects that had been discussed during the meeting, and to return to the next session with new experience-based knowledge. Participants became aware that some instructions in the guidelines from the National Board of Health were not fully up-to-date; the recommendations for congenital cataract, for example. The serial nature of the meetings meant that such questions of doubt could be checked in the interim and discussed at the next meeting.

#### Reflections and ethical considerations

The serial character of the meetings and the continual re-evaluation of the drafts (Fig. 1) made room for participants to further reflect between meetings on the topics discussed. During the first meeting, the idea that information collected at the mother’s antenatal care visits could automatically be transferred to the child’s health record was presented and calmly received in the group. However, at the third meeting an intense discussion arose concerning ethical issues raised by the idea of transferring certain kinds of information to the child’s record, e.g. alcohol abuse in the family. The serial application of the method provided time for important critical reflection on themes and, in this case, the ethical challenges around a potential transfer of data.

#### Adjustable template

Although the group did not succeed at the first meeting in producing a final model for the PCHE at five weeks, the NGT secured the production of a fruitful draft which was applied as a model for the first and all succeeding PCHEs (See Table 2). The flexibility of the NGT therefore led to the production of a template that could be used and tailored during the development of systematic electronic health records for all seven PCHEs. The template was based on the experience of frontline professionals, the guidelines from the National Board of Health, and best evidence. The findings were practice-near, experience-based, and therefore directly applicable to PCHE work in general practice.

## Discussion

Our main findings from working with the NGT relate to its flexibility and modifiability. The flexibility of the method confirmed its suitability for complex research questions, such as ours; while the production of an adjustable template with consensus results made the meetings’ outcomes both manageable and tangible. The functionality of the modified serial meeting design provided fruitful time for continued reflection on the results and previous discussions, as well as providing opportunities for relevant checks between meetings where a lack of knowledge or doubts had become apparent. The latter provided openings for systematic development of knowledge.

Some of our results concur with findings from previous projects working with the NGT and the model’s flexibility has been recognised by other studies that also successfully modified the original NGT design and experienced an improvement [5–10]. One study had difficulty with the ranking in step 5 (Table 1) [8], which we also report in our findings. They ended up voting when consensus could not be reached through ranking, while the present study chose to divide the panel into pairs to work with the themes, before returning once more to discussion in plenum. In line with our findings, other studies have also found that the original NGT structure, with one meeting allocated to reach final recommendations, was not sufficient for an in-depth elaboration of themes [8, 30]. The serial character of the meetings in this study is comparable to the Delphi method [12, 15] where consensus is obtained through evaluation of written documents that are sent back and forth among participants a number of times until consensus is reached. Therefore, in the present study we incorporated strength from the Delphi method into the modified version of the NGT.

Experiences emerged during the working processes that, to our knowledge, have not previously been reported by other studies. A central feature of using the NGT is that a question is investigated extensively from a broad spectrum of viewpoints and thereby creates awareness of overlaps, knowledge-sharing, gaps in knowledge, or unproductive working patterns. In this project, our participants became aware of the possibility of using existing knowledge about their patients, but also about the potential ethical downside of such a practice. By documenting all proposals and ideas, the NGT model ensures that no insights are lost through the potential uncertainty of some participants, while at the same time tangible products in the form of written documents are produced.

### Implication of findings for future research

#### Related to the specific research project

It is anticipated that the possibility of using electronic health records as a support when carrying out PCHEs in the future will systematise and develop both the structure and the content of the PCHEs in general practice. However, experience and intuition are fundamental and effective elements of everyday working life in general practice, not least when it comes to diagnosing children [27, 31, 32]. It is therefore crucial that the electronic health records do not compromise this practice, which is why the use of the records will be a supportive option and not a mandatory practice. Lippert et al. have pointed to a need for further discussion about the relationship between situatedness and standardisation in primary care and for further empirical investigations of the possible consequences of standardisation processes [33]. DanChild, of which the present study is a part, has a combined vision to investigate GPs’ responses to electronic health records as well as to develop child health through cohort research. While the NGT as a method encourages consensus and practice-near solutions, it is important to emphasise that the success of the electronic health records is dependent on a continued and reciprocal collaboration with general practice [34, 35].

#### Related to the applicability of the modified method

The modifications we made to the NGT were feasible and did not lose the method’s advantageous structure. We believe this was because the participants and the facilitator shared a common professional background as GPs, limiting the perspectives to one professional grouping. Participants had been asked to read relevant chapters in the Danish National Board of Health’s guidelines on PCHEs as well as a thematically relevant report, further enhancing a mutual starting point. However, we did not check whether or not they had read the documents. We felt that this would unnecessarily highlight the fact that one of the participants, RE, was also one of the project’s initiators. Therefore we cannot guarantee that all participants had a common starting point for discussion. Finally, the project had a well-defined goal, namely the production of content and a format for electronic health records supporting the PCHEs, and that concrete purpose enabled a softening of the original NGT model’s mechanical steps, without the group losing its focus. Based on our experience with the modified NGT all three aspects are crucial for future researchers implementing similar changes to the original NGT model.

### Strengths and limitations of the study

By asking highly professionally engaged GPs with a specific interest in the PCHEs to reach consensus and suggest a way forward for all GPs to follow, our results may be ambiguous for the average practitioner. The project group is aware of this risk and will incorporate it into their continued work with electronic health records by pilot testing the product and by giving individual practitioners flexibility to use the record in their own way.

In this project two of the article’s authors participated either as facilitator (KL) or as a member of the panel (RE), and they worked with the drafts between the meetings. Double roles like these are not uncommon in similar projects [8], but still worth critical reflection and consideration. Any data collection, analysis, and conclusion are inextricably entwined with the researcher’s presuppositions as well as the positions adopted while collecting the data [36, 37]. According to Skjervheim, researchers can only gain access to social phenomena of interest by recognising themselves as a contributing participant [38]. In this case, RE is an experienced GP with a known research interest in child health, and took part in the panel as an equal to the other participants. RE was, however, aware of her double role in the process and continuously reflected on the effect it might have on the way her suggestions and comments were received by the group, and, ultimately, on how it might have affected consensus. One could discuss if KL’s and RE’s work between the meetings minimised the democratic process, by giving them more influence than the rest of the group. This risk was reduced as much as possible, by the process of repeatedly evaluating their work in plenum at subsequent meetings. During these evaluations the other participants actively suggested critical corrections and this leads us to believe that the final product meets a satisfactory degree of representativeness. Furthermore, we attempted to minimise bias by enrolling co-authors who were not actively implicated in the data producing process. One of them even participated as an observer at one of the meetings. Finally, the authors recognise that objective knowledge in the form of *true* consensus is a naïve understanding of reality. Following Haraway, it might be more fruitful to think of knowledge as situated within a context [39]. While the point of view within a context has a more limited range than disembodied objectivity, situated points of view are richer in content as they take into account the numerous bits of information constituting the context and the environment of that point of view. For the present project, it means acknowledging the influence KL and RE had on consensus, while at the same time recognising this as a given condition that simultaneously supported the success of the group’s progressive work.

## Conclusions

To our knowledge, this study is the first to report on Danish GPs using the NGT to identify key areas of focus and to structure quality marker development in general practice. The structured interactive process used in this study supported equal opportunities for experienced professionals to significantly contribute to the development of electronic health records to support PCHEs in Danish general practice. By using the modified NGT, participating GPs actively expressed their views through structured discussions as a group, through working in pairs, and through the process of reaching final consensus. In accordance with previous studies [3] we therefore argue that the original NGT model developed in the late 1960s [5] can be modified advantageously and used to explore developmental work and changes in general practice. Due to the integration of experienced professionals from the very beginning of the process the results are practice-based and applicable. We are confident that the NGT model can be useful for capturing group perspectives in complex working areas such as general practice, and we recommend the NGT as a working tool in general practice development in the future.

## Abbreviations

ES: Elisabeth Søndergaard
Fig.: Figure
GPs: General practitioners
KL: Kirsten Lykke
NGT: The nominal group technique
PCHEs: Preventive child health examinations
RE: Ruth K. Ertmann

## Footnotes

1 Creating electronic health records is part of DanChild, a research and quality development project, with the goal to develop a new national birth cohort with potential to nest randomised trials in general practice. The cohort will support the development of knowledge about child health which is highly relevant to the work in general practice.
